# Glycyrrhizin Blocks the Detrimental Effects of HMGB1 on Cortical Neurogenesis after Traumatic Neuronal Injury

**DOI:** 10.3390/brainsci10100760

**Published:** 2020-10-21

**Authors:** Susruta Manivannan, Balkis Harari, Maryam Muzaffar, Omar Elalfy, Sameera Hettipathirannahelage, Zoe James, Feras Sharouf, Chloe Ormonde, Mouhamed Alsaqati, William Gray, Malik Zaben

**Affiliations:** 1 Neuroscience and Mental Health Research Institute, Hadyn Ellis Building, Cathays, Cardiff CF24 4HQ, UK; manivannansusruta@gmail.com (S.M.); HarariB@cardiff.ac.uk (B.H.); MuzaffarMA@cardiff.ac.uk (M.M.); omarelalfy@me.com (O.E.); sameera_he@hotmail.co.uk (S.H.); zoz364@googlemail.com (Z.J.); SharoufFH@cardiff.ac.uk (F.S.); OrmondeCE@cardiff.ac.uk (C.O.); alsaqatim@cardiff.ac.uk (M.A.); graywp@cardiff.ac.uk (W.G.); 2 Division of Psychological Medicine and Clinical Neurosciences (DPMCN), School of Medicine, Cardiff University, Cardiff CF24 4HQ, UK

**Keywords:** traumatic brain injury, neurogenesis, HMGB1, neuroinflammation

## Abstract

Despite medical advances, neurological recovery after severe traumatic brain injury (TBI) remains poor. Elevated levels of high mobility group box protein-1 (HMGB1) are associated with poor outcomes; likely via interaction with receptors for advanced-glycation-end-products (RAGE). We examined the hypothesis that HMGB1 post-TBI is anti-neurogenic and whether this is pharmacologically reversible. Post-natal rat cortical mixed neuro-glial cell cultures were subjected to needle-scratch injury and examined for HMGB1-activation/neuroinflammation. HMGB1-related genes/networks were examined using genome-wide RNA-seq studies in cortical perilesional tissue samples from adult mice. Post-natal rat cortical neural stem/progenitor cell cultures were generated to quantify effects of injury-condition medium (ICM) on neurogenesis with/without RAGE antagonist glycyrrhizin. Needle-injury upregulated TNF-α/NOS-2 mRNA-expressions at 6 h, increased proportions of activated microglia, and caused neuronal loss at 24 h. Transcriptome analysis revealed activation of HMGB1 pathway genes/canonical pathways in vivo at 24 h. A 50% increase in HMGB1 protein expression, and nuclear-to-cytoplasmic translocation of HMGB1 in neurons and microglia at 24 h post-injury was demonstrated in vitro. ICM reduced total numbers/proportions of neuronal cells, but reversed by 0.5 μM glycyrrhizin. HMGB1 is activated following in vivo post mechanical injury, and glycyrrhizin alleviates detrimental effects of ICM on cortical neurogenesis. Our findings highlight glycyrrhizin as a potential therapeutic agent post-TBI.

## 1. Introduction

Traumatic brain injury (TBI) is a global public health problem; predicted by the World Health Organisation (WHO) to become one of the leading causes of death and disability by the end of this year [1,2]. The best available medical and neurosurgical interventions reduce mortality rates after severe TBI, but a significant number of survivors are left with life changing moderate-to-severe disabilities [3]; the negative effects are largely related to sensory-motor, neuro-cognitive, behavioural and memory deficits [4,5,6,7,8,9]. From a pathophysiological standpoint, TBI causes significant neuronal cell death primarily in the cerebral cortex [10], largely evident during the secondary stages of host response to injury [11]. Remarkably, this is associated with surprisingly high levels of neural stem/progenitor cell (NSPC) proliferation in the peri-lesional cortex, indicating the brain’s attempts to self-repair [12]. Newly born neurons however largely die before developing into mature, functioning neurons [13], an observation which may in-principle explain the lack of meaningful functional recovery post-TBI.

Neuroinflammation constitutes a significant part of the mechanisms involved in the secondary brain response to injury. The response of the cortical NSPC niche to the change in the surrounding microenvironment, dominated by the abundance of inflammatory cytokines, is a complex phenomenon [14]. Understanding this interaction in the context of TBI is, thus, paramount for identifying therapeutic strategies for achieving functional neural regeneration post-TBI and potentially improving clinical outcomes. Attention has been directed recently towards the high mobility group box protein 1 (HMGB1); a pivotal inflammatory cytokine released acutely following TBI. HMGB1 acts as a non-histone DNA binding protein under physiological conditions but is released passively by necrotic neurons or secreted actively by immune cells as an “alarmin” under pathological insult [15]. HMGB1 has been shown to mediate brain damage through interactions with receptors for advanced glycation end products (RAGE) and Toll-like receptors 2/4 (TLR2/4) in various animal models of TBI [16,17]. Consistently, high serum and cerebrospinal fluid (CSF) levels of HMGB1 in patients with TBI are associated with poor clinical outcomes [18,19,20]. However, the role of HMGB1 in neurogenesis and brain repair following TBI has yet to be fully explored. 

We and others have previously demonstrated that the rodent cortex harbours quiescent endogenous multipotent stem cells that proliferate in vitro following TBI [21,22]. We, herein, expand those findings to demonstrate that sterile needle cortical injury activates the canonical pathways of the pro-inflammatory cytokine HMGB1 at the level of the transcriptome in vivo, and enhances its sustained activation in microglia and neuronal cells in vitro. We further show that mixed-neuro-glia injury conditioned medium has a detrimental effect on cortical neurogenesis; the effect is pharmacologically reversible by the naturally occurring HMGB1 antagonist glycyrrhizin, likely through interactions with RAGE. Our findings, for the first time, implicate the HMGB1-RAGE axis in inhibiting post-injury cortical regeneration, and highlight glycyrrhizin as a potential drug to enhance brain repair after TBI.

## 2. Materials and Methods

**Figure 1:**
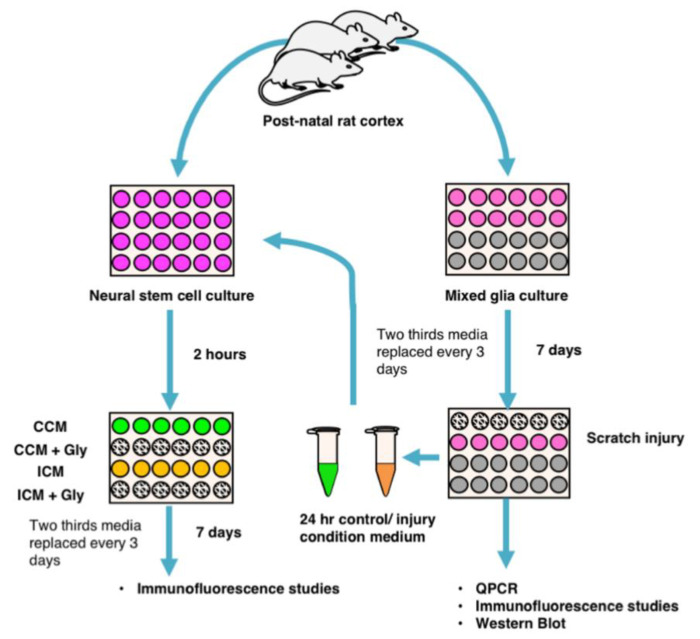
Schematic diagram of the experimental design. Mixed glial and neural stem cell cultures were generated from the post-natal rat cortex (described in detail in the text). Gene expression, immunofluorescence, and western blot studies were performed on mixed glial cultures at multiple time points, post injury. Abbreviations: CCM—control condition media, Gly—glycyrrhizin, ICM—injury condition media.

All animal experiments and surgical procedures were conducted under the UK Animals (Scientific Procedures) Act 1986, and subjected to local ethical review and relevant personal project licence (P8157151A) as outlined by Cardiff University School of Psychology. All attempts were made to ensure the comfort and respect of the animals. The animals were kept in a controlled environment maintained with a 12-h light/dark cycle. Rats had access to food and water ad libitum. Rat cortical NSPC were generated from post-natal Sprague Dawley rats (P7–10 days old, Figure 1).

### 2.1. Generating Mixed Glial Cultures and Injury Condition Media

Mixed glial cultures were generated from the post-natal cortices of wild type Sprague-Dawley rats. Cells were then plated on glass coverslips coated with Poly-L-Lysine to a density of 200,000 cells per mL in a 24-well plate. Cultures were grown for 7 days in a culture medium of Neurobasal A (NBA), 1% B27 (neuronal cell culture supplement) and 0.5 mM glutamine, 1% penicillin/streptomycin and 10% foetal bovine serum (FBS). Two-thirds of the culture medium was replaced with fresh medium every 2–4 days. At 7 days in vitro (DIV), the culture media was replaced with serum-free media (NBA, 1% B27 and 0.5 mM glutamine), and mixed glial cultures were subjected to scratch injury with a 20 gauge venepuncture needle (as described elsewhere, [23,24]). Scratch injury was performed with adherence to a 2 × 2 mm grid pattern positioned beneath the plate. Injury (ICM) and control condition media (CCM) were collected and stored in the freezer (−80 °C) until subsequent use.

### 2.2. Generating NSPC Cultures

NSPC cultures were generated from the postnatal Sprague–Dawley rat cortex (P7–10) as described previously [25]. Briefly, using sterile conditions, cortices were quickly dissected and cut on a McIlwain tissue chopper into 400 µm-thick slices in Gey’s balanced salt solution (Life Technologies, Paisley, UK), supplemented with 4.5 mg/mL glucose at 4 °C. The tissue slices were then digested with 2 mg/mL papain (lypophilised powder > 10 units/mg protein; Sigma, Gillingham, Dorset, UK) in pre-warmed Neurobasal A (NBA), supplemented with 2% B27 (Thermo Fisher Scientific, Paisley, Scotland, UK) and 0.5 mM glutamine (Sigma, Gillingham, Dorset, UK) for 30 min at 37 °C. After washing, cell release was achieved by trituration. To remove cell debris, NSPCs were purified and enriched using a 2-step density OptiPrep (Axa-shields, Oslo, Norway) gradient. Viable cells were then seeded at a density of 100,000 cells per ml in a culture media and plated directly onto 24-well plates containing poly-L-lysine coated glass coverslips. The cells were incubated at 37 °C with 5% CO_2_ and 95% humidity. At 2 h post-plating, the culture medium was refreshed and experimental conditions were established as follows: CCM (1:1), ICM (1:1), glycyrrhizin, CCM plus glycyrrhizin or ICM plus glycyrrhizin. In vivo kinetics of glycyrrhizin indicated that the therapeutic concentration of glycyrrhizin lay between 0.4 and 1.0 μM [26], therefore we used a concentration of 0.5 μM. Glycyrrhizin powder was dissolved directly into the culture medium to achieve the appropriate concentration. Two-thirds of the culture medium was replaced every 3 days. Cultures were not expanded using mitogens prior to experimental use, but were used immediately after generation before rapidly dividing precursors became dominant, so that they were more representative of the mixed complex in vivo situation.

### 2.3. Immunocytochemistry

Immunofluorescent staining was performed on 4% paraformaldehyde (PFA)-fixed cells as described by our research group previously [25]. The following primary antibodies were used in phosphate-buffered saline with 0.1% Tween detergent (PBS-T-0.1%)/ Triton X-100: mouse anti-rat Nestin 1:1000 (Sigma, Gillingham, Dorset, UK), mouse anti-GFAP (glial fibrillary acid protein) 1:1000 (Sigma, Gillingham, Dorset, UK), rabbit anti-GFAP 1:1000 (Agilent Dako, Cheshire, UK), mouse anti-Tuj1 1:1000 (Sigma, Gillingham, Dorset, UK), rabbit anti-TuJ1 1:1000 (Abcam, Cambridge, UK), mouse anti-RAGE receptor (Abcam, Cambridge, UK), anti-IB4 (isolectin B4) (Thermo Fisher Scientific, Paisley, UK), anti-HMGB1 (Abcam, Cambridge, UK) and rabbit anti Sox2 1:500 (Abcam, Cambridge, UK). Primary antibodies were probed using Alexa Fluor^®^ 488 or Alexa Fluor^®^ 555-conjugated anti-rat 1:1000, anti-mouse 1:1000, anti-Goat 1:1000 and/or anti-rabbit 1:500 secondary antibodies (Thermo Fisher Scientific, Paisley, UK). Cells were then counterstained with the nuclear stain 4′,6-diamidino-2-phenylindole (DAPI; 5 µg/mL) (Sigma, Gillingham, Dorset, UK).

### 2.4. Cell Images and Quantification

Imaging of cells in culture was performed on a Leica DM6000B Upright System microscope (Fisher Scientific, Leicestershire, UK). The area of a 20× field was measured using a 255 µm grid graticule slide (Microbrightfield, Williston, VT, USA). Cell counting was performed on six random fields per well using ImageJ software [27]. Raw data from the field counts were averaged and plotted ± standard error mean (SEM) and expressed as cells/mm² per well, based on a sample of four to eight wells per condition per experiment. All experiments were repeated at least three times. One experiment consisted of two–four cortices from two–six animals, pooled and prepared as described above. The following parameters were identified: total cell count (DAPI^+^ cells), neuronal cell count (Tuj1^+^ cells), neurogenesis (%Tuj1/DAPI). When quantifying proportions of cells with cytoplasmic HMGB1, each cell subtype (Tuj1^+^, GFAP^+^ and IB4^+^) was counted, and the distribution of HMGB1 was characterised as nuclear or cytoplasmic [28]. The number of cells with cytoplasmic HMGB1 was divided by the total number of cell subtypes to yield the proportion of cytoplasmic HMGB1 in each cell phenotype. Data were plotted using the GraphPad Prism software (Version 8.0.1, GraphPad Software Inc. San Diego, CA, USA) and mean percentage values were statistically analysed using the unpaired Student’s *t*-test for 2-group comparisons, and one-way ANOVA or two-way ANOVA for multiple comparisons as indicated (values *p* < 0.05 were considered significant).

### 2.5. Transcriptome Analysis

All animal experiments and surgical procedures were conducted under the UK Animals (Scientific Procedures) Act 1986, and subjected to local ethical review and relevant personal, project and institutional licenses. A total of 14 C57Bl6 mice were included in this study, aged 9 months (± one week) and weighing 25–30 g. All surgical procedures were performed under isoflurane anaesthesia induced in an induction chamber with 5% isoflurane in oxygen at 0.8 l/min. Anaesthesia was maintained by passive inhalation of isoflurane (1.5–2.5%) in a mixture of oxygen (0.8 l/min). Each mouse received a unilateral penetrating needle injury (26 gauge) to the cerebral cortex and as deep as the striatum. A subcutaneous injection of 0.5 mL saline glucose was administered in addition to 0.05 mg/kg buprenorphine using 300 µL insulin syringes before transfer to a warm recovery chamber. Tissue was collected at 0 h (no needle insertion), 1 h and 24 h post needle injury for bulk RNA sequencing, following schedule 1. A 3 mm^3^ cube core of tissue around the injury site was collected using a mouse brain matrix and stored in −70 °C. Snap chilled brains were immediately homogenized in TRIzol^TM^. Total RNA was extracted with TRIzol^TM^ according to the manufacturer’s recommendations (Thermo Fisher Scientific, Paisley, UK) and the tissue integrity was confirmed on an Agilent Bioanalyzer (Agilent Technologies, Cheshire, UK).

Paired-end reads from Illumina sequencing were trimmed with Trim Galore [29] and assessed for quality using FastQC Babraham [30] and default parameters. Reads were mapped to the mouse GRCm38 reference genome using STAR [31] and counts were assigned to transcripts using FeatureCounts [32] with the GRCm38.84 Ensembl gene build GTF (gene, transcript, and exon coordinates). Both the reference genome and GTF were downloaded from the Ensembl FTP site [33]. Differential gene expression analyses were performed using the DESeq2 package [34]. Genes were discarded from the analysis if differential expression failed to be significant (significance: adj.pval 0.05, Benjamini–Hochberg correction for multiple testing). Differential gene splicing analyses used the DEXSeq package [35] (significance: adj.pval < 0.05, Benjamini–Hochberg correction for multiple testing).

We evaluated the expression of genes associated with HMGB1 signaling using the ingenuity pathway analysis (IPA) program [36]. All relevant genes that exhibited a significant log2fold change (differentially expressed genes) were also pooled across all time lines. Using Morpheus, fragments per kilobase of exon per million (FPKMs) [37] were clustered for all differentially expressed genes associated with HMGB1 signaling and a heatmap with hierarchal clustering was obtained.

### 2.6. PCR Assay

For PCR, total RNA was extracted from cultured cells using the GenElute Mammalian Total RNA Miniprep kit (Sigma, Gillingham, Dorset, UK) and directly reverse-transcribed to complementary DNA (cDNA) by using a miScript II RT Kit (50) (Qiagen, Crawley, Manchester, UK). The cDNA was then amplified using a one-step PCR kit (Rat Custom real-time PCR assay for use with SYBRgreen chemistry) (PrimerDesign Ltd., Eastleigh, UK) in a real-time thermocycler (Rotor-Gene 6000, Corbett Robotics. Ltd, Cambridge, UK). The PCR reaction amplification conditions were: enzyme activation for 10 min at 95 °C followed by 15 s for denaturation at 95 °C and then the data was collected in 60 s at 60 °C. Cytokine primers were: HMGB1; forward: AGTCCCAGCGAAGGCTATCA and reverse: CAGTCAAGTTTCCTGAGCAATCC; NOS2; forward: CACCACCCTCCTTGTTCAAC and reverse: CAATCCACAACTCGCTCCAA; TNF-alpha; forward: GCTCCCTCTCATCAGTTCCA and reverse: CTCCTCTGCTTGGTGGTTTG (PrimerDesign Ltd., Eastleigh, UK). Results were normalized against the housekeeping gene β-actin. Quantitative PCR was performed with 1 µL of the reconstituted primer mix (0.2 µM), 10 µL of PrimerDesign 2X PrecisionTM MasterMix with SYBRgreen and 4 µL PCR-Grade water, to which 25 ng/µL of the indicated cDNA (control or injury at different time points post-injury) was added to a final volume of 20 µL. Fluorescent data was collected at least once during each cycle of amplification, which allowed us to perform real-time monitoring of the amplification. Data was automatically normalized and a threshold was set at the level where the rate of amplification was the greatest during the exponential phase. As soon as the cycle threshold (Ct) values were collected, raw data was processed and analysed using the comparative Ct method (2[-Delta Delta C(t)] method (2[-ΔΔCt])) [38], where the comparative expression level equals 2-ΔΔCt. A validation experiment confirmed that the amplicons performed equally efficiently across the range of the initial template amounts [39].

### 2.7. Western Blotting

Cultured cells were washed with ice-cold PBS and their adherence disrupted using a cell scraper. The cells were then treated with ice-cold radioimmunoprecipitation assay (RIPA) lysis buffer (Sigma, Gillingham, Dorset, UK) and protease inhibitor cocktail (Sigma, Gillingham, Dorset, UK) and agitated for 30 min at 4 °C. Cells were centrifuged at 21,000 rcf (relative centrifugal force) at 4 °C and the supernatant was collected. The supernatant was treated with appropriate amounts of lithium dodecyl sulphate (LDS) sample buffer (NuPAGE) and sample reducing agent (NuPAGE), then samples were heated at 95 °C for 5 min. Electrophoresis was carried out using the Bolt 4–12% Bis–Tris Plus Gels (Life Technologies, Paisley, UK) with 15 µg of protein loaded per sample. Samples were transferred to nitrocellulose membranes and these blots were incubated in a blocking solution 5% (*w/v*) powdered milk in Tris-buffered saline containing 0.1% (v/v) Tween 20 (TBS-T) for 60 min, at RT. Blots were then incubated overnight at 4 °C with a primary antibody against HMGB1 (1:500) (ab77302, Abcam, Cambridge, UK), diluted in the blocking solution. After washing in TBS-T, blots were incubated with an appropriate IRDye^®^-conjugated secondary antibody (LI-COR Biosciences Ltd, Cambridge, UK). Proteins were visualised using the LI-COR/Odyssey infrared imaging system (LI-COR Biosciences Ltd, Cambridge, UK). Bands were analysed by densitometry using the Odyssey application software and expressed as the density of the protein of interest to that of GAPDH (glyceraldehyde 3-phosphate dehydrogenase), and normalised to the control.

### 2.8. Statistical Analysis

Data were plotted using the Prism software (GraphPad Software Inc. Version 8.0.1, San Diego, CA, USA) and mean percentage values were statistically analysed using the unpaired Student’s *t*-test or one- and two-way ANOVA for multiple comparisons as appropriate (where values *p* < 0.05 were considered significant.)

## 3. Results

### 3.1. Mechanical Scratch Injury Enhances Microglial Activation and Neuronal Cell Loss In-Vitro

**Figure 2:**
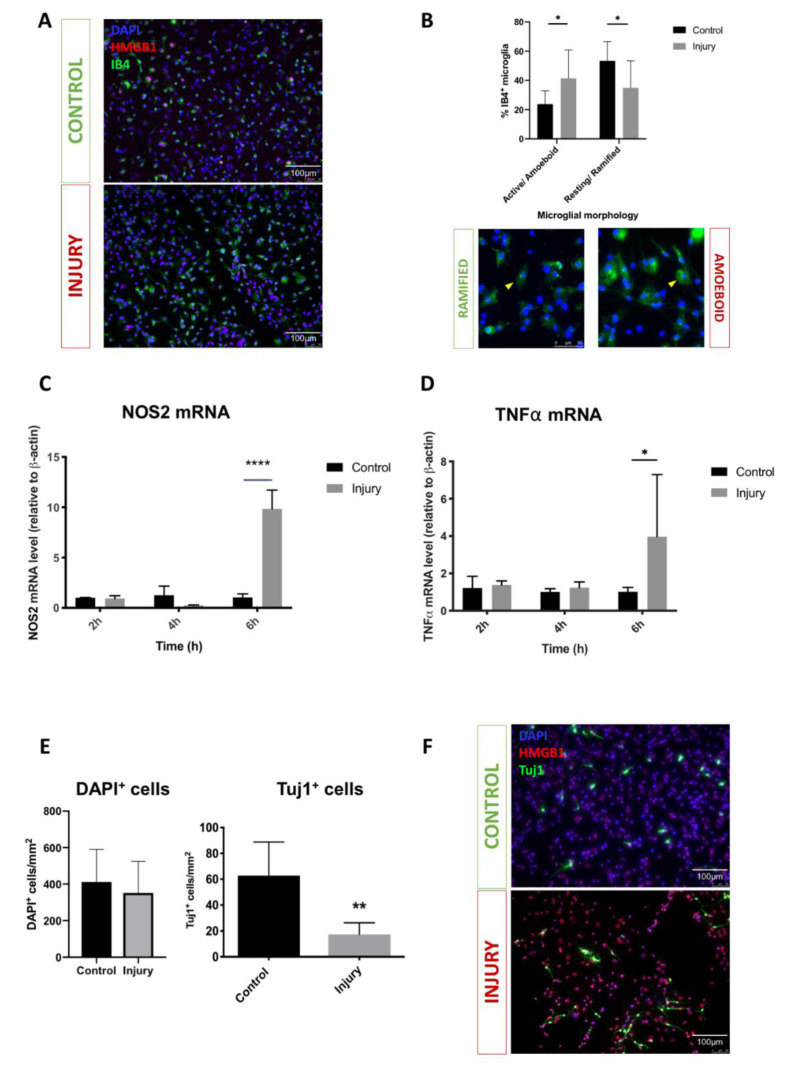
Mechanical injury of cortical mixed glial cultures enhanced microglial activation and upregulated pro-inflammatory cytokine expression. (**A**) Representative immunofluorescence microscopy images of microglia in control and injury mixed glial cultures (IB4—green, DAPI—blue, HMGB1—red); (**B**) Injury significantly increased proportions of microglia with active/amoeboid (* *p* < 0.05) and reduced proportions of microglia with resting/ramified (* *p* < 0.05) morphologies, representative fluorescent microscopy images of different microglial morphologies (yellow arrows); (**C**,**D**) Significant upregulation of pro-inflammatory cytokine expression demonstrated by an acute increase in NOS-2 (**** *p* < 0.0001) and TNF-alpha mRNA levels (* *p* < 0.05); (**E**) Significant loss of neurons (Tuj1^+^ cells) (** *p* < 0.01) but no significant difference in total cell count (DAPI^+^ cells) at 24 h post-injury; (**F**) Representative immunofluorescence microscopy images of neurons in control and injury mixed glial cultures (Tuj1—green, DAPI—blue, HMGB1—red). Scale bars 100 µm, 20× magnification in microscopy images. The subfigures (**B**–**D**) show two-way ANOVA and the subfigure (**E**) shows the unpaired *t*-test used for statistical analysis with *p* < 0.05 considered significant. *n*: 3–4 animals, four wells per condition and three repeats per experiment. Abbreviations: HMGB1—high mobility group box protein-1

Cortical mixed neuro-glial cell cultures (7DIV) were subjected to standard scratch injury as described elsewhere [23,24], Figure 2A. Post-injury microglia activation was examined by quantifying levels of expression of the mRNA of two key pro-inflammatory cytokines (*TNF-α* and *NOS-2*) [40], and examining microglia morphology utilising IB-4 staining [41]. Consistent with previous studies we observed significant upregulation in *TNF- α* and *NOS-2* mRNAs expression at 6 h post-injury (4.0-fold change, *p* = 0.02; 9.8-fold change, *p* < 0.0001, respectively; Figure 2C,D, indicating glial activation). Consistently, our immunohistochemistry studies revealed a significant increase in proportions of IB4^+^ cells displaying an active/amoeboid morphology (23.7 vs. 41.2%; *p* = 0.02) and a reduction in resting/ramified morphology (53.4 vs. 34.9%; *p* = 0.01), Figure 2B. To examine whether this injury-induced inflammatory response is detrimental to neuronal cells, we quantified the numbers of neuronal cells (Tuj1^+^) in cultures at 24 h post-injury. Not surprisingly, injury-induced inflammation was associated with a significant decrease in the number of Tuj1^+^ cells from 62.8 ± 11.6 to 17.4 ± 4.0 cells/mm^2^ *p* = 0.0061, Figure 2E.

### 3.2. Transcriptome Analysis Reveals Needle Cortical Injury Upregulate HMGB1 Related Networks In-Vivo

**Figure 3:**
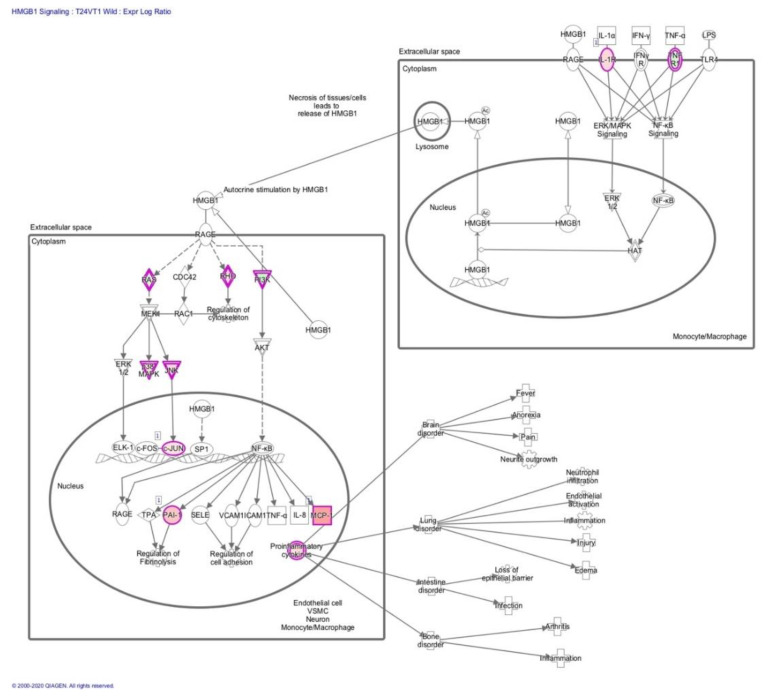
Schematic of the HMGB1 signaling pathway. The HMGB1 signaling pathway as derived from the IPA analysis results of RNA transcriptomics.

**Figure 4:**
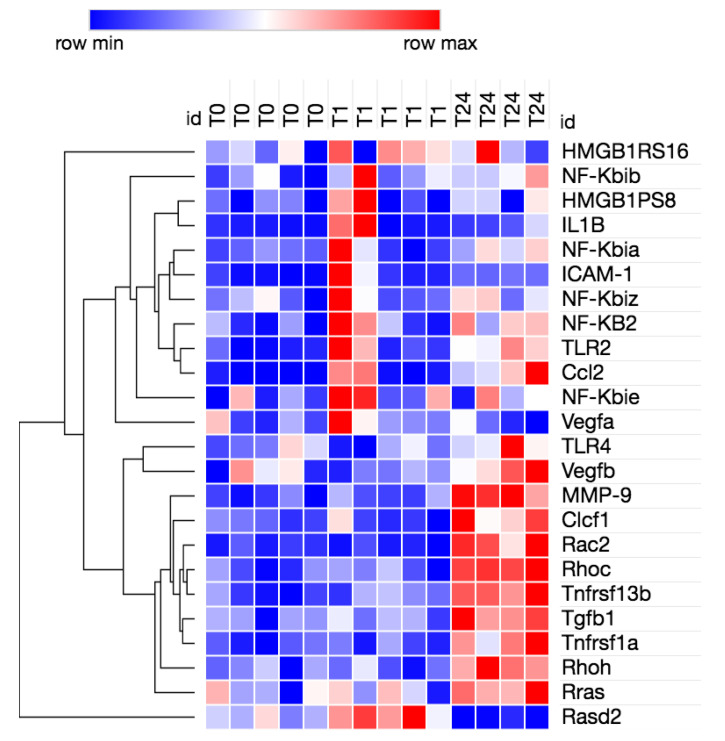
Upregulation of HMGB1 signaling pathways at 1 h and 24 h post needle injury. The heatmap with horizontal hierarchal clustering depicts differentially expressed genes (fragments per kilobase of exon per million—FPKM) associated with HMGB1 signaling across all time points (0 h: no injury, 1 h post-injury and 24 h post-injury). The red colour denotes upregulation of a molecule in HMGB1, signaling cascade, whilst the blue colour indicates downregulation of a molecule. Gene symbols with a single border represent single genes. Double borders represent a complex of genes or the possibility that alternative genes might act in the pathway. The node shapes denote enzymes, phosphatases, kinases, peptidases, G-protein coupled receptor, transmembrane receptor, cytokines, and other. The gradient colour degree means a slightly different expression tendency of that molecule, dark red > 2 fold change, light red 1–2 fold change, *n*: 4–5 per condition.

**Figure 5:**
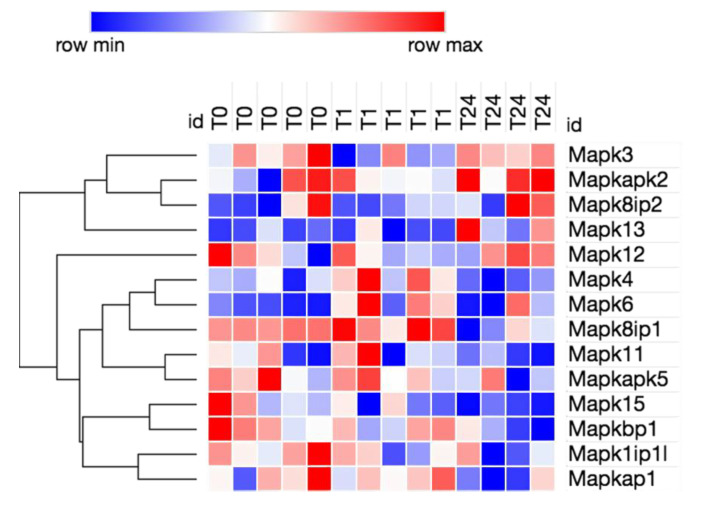
Changes in mitogen-activated protein kinase (MAPK) isoforms signaling at 1 h and 24 h post needle injury. The heatmap with horizontal hierarchal clustering depicts differentially expressed genes (FPKM) associated with HMGB1 signaling across all time points (0 h (no injury), 1 h post-injury and 24 h post-injury). The red colour denotes upregulation of a molecule involved in the HMGB1 signaling cascade, whilst the blue colour indicates downregulation.

In a mouse model of needle penetration brain injury, we analysed the 1 h and 24 h neuroinflammation transcriptomes to determine whether HMGB1 signaling pathways are coordinately regulated in the peri-lesional cortical tissue after injury. We used Bioinformatics Resources to gain insight into the potential contribution of HMGB1 into the neuro-inflammatory biological processes enriched in common injury-induced pathways. HMGB1 emerged as one of the top upregulated canonical pathways at 1 h and 24 h post-injury time points whilst injury differentially regulated the major categories of the HMGB1 activation intracellular canonical pathways, Table 1, Figure 3, Figure 4 and Figure 5. To corroborate these functional findings, we analysed the influence of co-action on molecular signaling networks post-injury using knowledge-based IPA [42]. Commonly induced gene sets revealed 8 signaling networks related to HMGB1 neuro-inflammatory pathway activation (Figure 3). In particular, the HMGB1 downstream transcription factor Nuclear Factor-κB (NFκB) was among the central modulator hubs (upregulation of IL1-R, TNFR1, NF-KB2, HMGB1RS16, NF-KB2, NF-KBIZ, IL1-B, TNFRSF11B, TNFRSF1A, TNFRSF13b, and TLR4, Figure 3 and Figure 4). Together, this data implies that HMGB1 pathways may induce the gene expression related to post traumatic cortical injury inflammatory response.

### 3.3. HMGB1 Cellular Expression and Release Are Increased after Mechanical Scratch Injury In-Vitro

**Figure 6:**
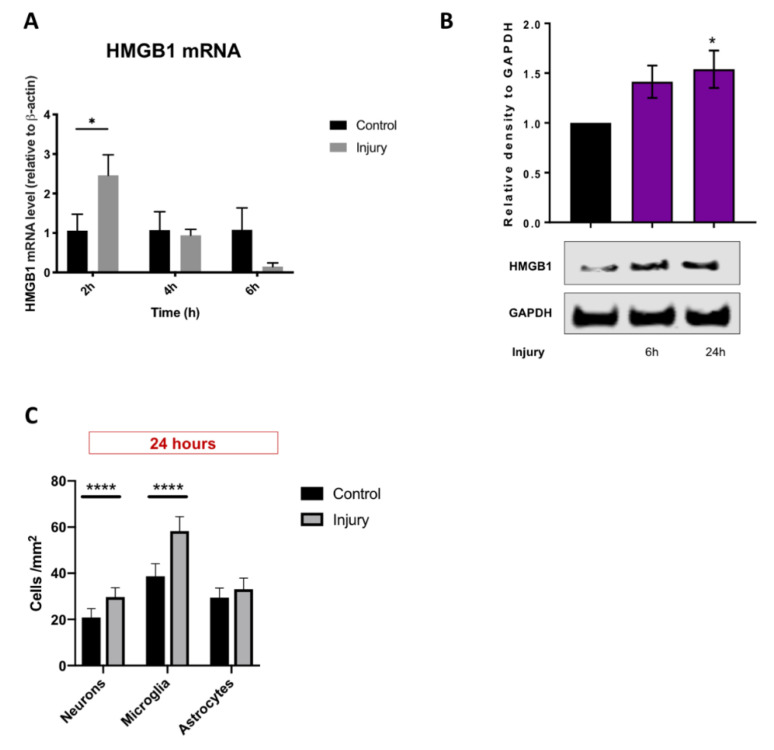
HMGB1 cellular expression and release are increased after mechanical scratch injury of rat cortical mixed glial cell cultures. (**A**) HMGB1 mRNA expression was significantly elevated as early as 2 h post-injury when compared to the control (* *p* < 0.05); (**B**) HMGB1 protein levels significantly increased at 24 h post-injury relative to the control on the western blot (* *p* < 0.05); (**C**) Percentage HMGB1 cytoplasmic translocation was significantly increased in both neurons (Tuj1^+^ cells) and microglia (IB4^+^ cells) at 24 h post-injury (**** *p* < 0.0001), but not in astrocytes (GFAP^+^ cells). Subfigures (**A**,**C**) show two-way ANOVA and (**B**) one-way ANOVA used for statistical analysis with *p* < 0.05 considered significant. *n*: 3–4 animals, four wells per condition and 3 repeats per experiment.

Cortical mixed neuro-glial cell cultures (7DIV) were subjected to standard scratch injury as described above before being proceeded for HMGB1 mRNA and protein expression. Interestingly, we observed a hyperacute significant upregulation in HMGB1 mRNA as early as 2 h post-injury (2.5-fold change; *p* < 0.05), Figure 6A. We examined whether injury-induced HMGB1 mRNA hyper-expression translates to sustained HMGB1 activation by quantifying the levels of HMGB1 protein using western blot. We observed an almost linear increase in HMGB1 release during the first 24 h post-injury, Figure 6B. We then examined HMGB1 activation by quantifying immuno-histochemically HMGB1 cellular sublocalisation at 24 h post-injury, Figure 6C [43]. Examining the subcellular differential contribution to HMGB1 post-injury activation, we observed a significant increase in the proportions of microglia (58.3 vs. 38.7%; *p* < 0.0001) and neurons (29.7 vs. 20.8%; *p* < 0.0001), but not astrocytes that have translocated HMGB1 from the nuclei to cytoplasm, Figure 6C. Taken together, these observations demonstrate hyperacute and sustained HMGB1 activation post-injury; with different temporal differential cellular subtype contribution.

### 3.4. Injury Conditioned Media Has a Detrimental Effect on Neuronal Progenitor Cell Survival and Differentiation

**Figure 7:**
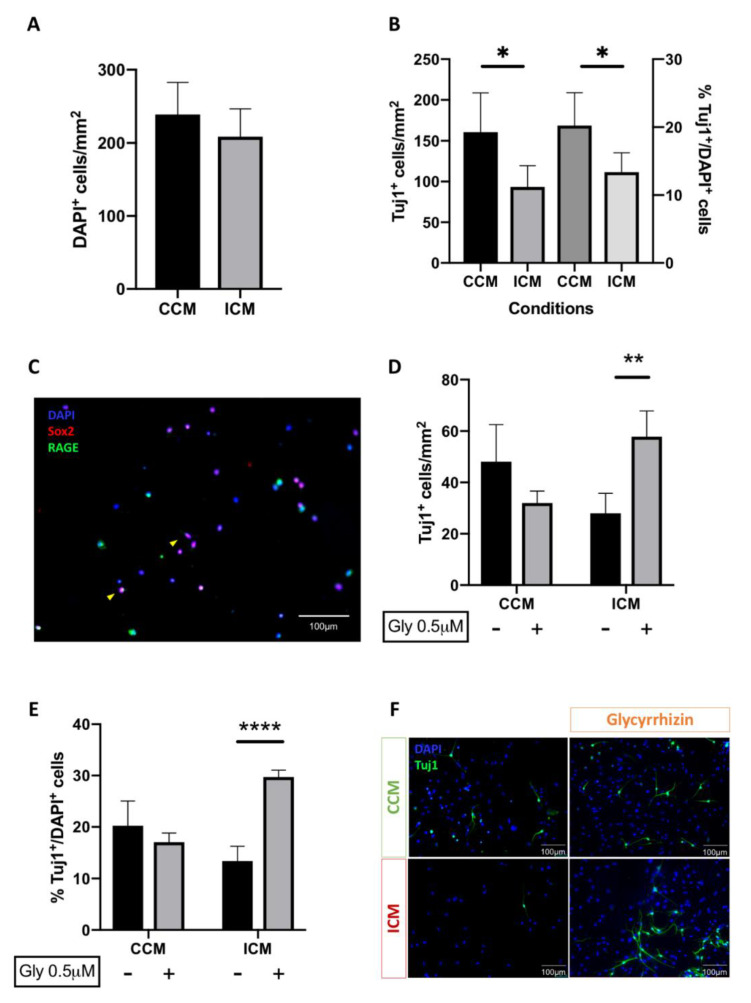
Injury conditioned media (ICM) has a detrimental effect on neuronal progenitor cell survival and differentiation, via a RAGE-dependent mechanism. (**A**) Exposure to ICM does not result in a significant change in total cell count; (**B**) ICM exposure results in a significant decrease in both neuronal cell survival (Tuj1^+^ cells/mm2; * *p* < 0.05) and differentiation (%Tuj1/DAPI cells; * *p* < 0.05); (**C**) Representative immunofluorescence microscopy images of rat cortical neural stem cell/progenitor cell cultures demonstrate RAGE expression in Sox2^+^ cells (yellow arrows); (**D**,**E**) Addition of glycyrrhizin, a RAGE antagonist, rescued the anti-neurogenic effects seen with addition of ICM (** *p* < 0.01, **** *p* < 0.0001); (**F**) Representative immunofluorescence microscopy images of rat cortical neural stem cell/progenitor cell cultures exposed to CCM and ICM in the absence or presence of glycyrrhizin (Tuj1- green, DAPI- blue). Scale bars 100 µm, 20× magnification in microscopy images. Two-way ANOVA used for statistical analysis with *p* < 0.05 considered significant. *n*: 3–4 animals, four wells per condition and 2 repeats per experiment. Abbreviations: CCM- control condition media; ICM—injury condition media.

Having demonstrated sustained activation of neuroinflammation in mixed-neuro-glia cell cultures post-injury; we next examined the effect of injury-conditioned medium (ICM) on neuronal stem cell survival and differentiation. In this set of experiments, postnatal (P7–12) rat cortical NSPC cultures were exposed to ICM or control conditioned media (CCM) for 5DIV before being processed for the raw counts and proportions of neuronal cells (TuJ1^+^). Exposure of NSPCs to ICM resulted in a significant decrease not only in the total neuronal cell counts (160.6 vs. 93.3 Tuj1^+^ cells/mm^2^; *p* = 0.013) but also the proportions of neuronal (Tuj1^+^) with respect to total cell count (DAPI) (20.2 vs. 13.4%; *p* = 0.013), Figure 7B. These observations clearly implicate that ICM has a detrimental effect on cortical NSPC survival and differentiation.

### 3.5. Blockade of HMGB1 Receptor Subtype RAGE Mediates ICM Anti-Neurogenic Effects

Given that we demonstrated HMGB1 is released acutely following injury and present in ICM, we examined the hypothesis that it hinders endogenous neural regeneration following injury. We first examined putative expression of the three main HMGB1 receptor subtypes by primitive cortical NSPCs using immunohistochemistry. Cortical neural progenitor cell cultures were harvested and fixed at 24 h. Cells were then immunohistochemically processed for the co-expression of the neural stem cell marker Sox2 and the HMGB1 target receptor RAGE. We observed consistent co-expression of the three receptors by Sox2-expressing cells raising the question regarding potential implication in neural stem cell ontology, Figure 7C.

We then selectively blocked the RAGE receptor in the presence or absence of ICM. Interestingly, the numbers (27.9 vs. 57.8 Tuj1^+^ cells/mm^2^; *p* = 0.002) and proportions of neurons (13.4 vs. 29.7%; *p* < 0.0001) under injury conditions increased significantly when cells were co-treated with glycyrrhizin, Figure 7D–F. This effect was not seen with addition of glycyrrhizin to cultures grown in CCM (20.2 vs. 17.1%; non significant (NS)). Therefore, addition of glycyrrhizin to NSPC cultures demonstrates a significant enhancement of neurogenesis under injury conditions alone, implicating a RAGE-dependent negative effect of HMGB1 on neurogenesis post-injury.

## 4. Discussion

In this study, we hypothesised that HMGB1 has an anti-neurogenic effect following TBI, given its crucial involvement in post-injury neuroinflammation. We validated an existing protocol to generate NSPCs from rat cortical tissue and used a needle scratch injury as an established model of in vitro TBI to study the effects of secondary injury. Importantly, we have demonstrated that: (i) HMGB1 gene expression is upregulated hyper-acutely following injury, it is released extracellularly, and associated with microglial activation; (ii) injury condition media has anti-neurogenic effects on NSPC cultures; and (iii) this effect is reversible with the pharmacological inhibition of HMBG1 at the target RAGE receptors, which are expressed by NSPCs.

Following the failure of various pharmacological strategies to prevent cellular death post-TBl [44,45], increasing attention has been directed towards the potential use of NSPCs for achieving regeneration and functional recovery. The question of whether stem cell transplantation or supporting endogenous neurogenesis is the optimal approach to improving outcomes remains to be answered. Conventionally, adult neurogenesis was thought to be confined to limited anatomical regions in the brain, namely the subventricular and subgranular zones [46]. In contrast, increasing evidence demonstrates the existence of NSPCs at the site of cortical injury in in vivo animal models of TBI [13,47,48]). Whilst it is unclear whether these cells are resident or migrating, it raises the exciting prospect of neural repair in a condition as anatomically heterogeneous as TBI [49]. However, existing studies demonstrate that immature newborn neurons do not go on to survive in the injured brain [13,50,51]. Therefore, optimising the micro-environment post-injury is paramount for both exogenous stem cell transplantation strategies and endogenous neural regeneration in order to achieve maturation of newborn neurons and functional integration. Also, understanding the specific relationship between neuro-inflammation and neurogenesis is of particular importance, and will avoid the generalisation of neuro-inflammation as an entirely deleterious process.

We used a well-established model of mechanical injury [23,24], to examine the dynamics of HMGB1 activation across different cell types. Scratch injury was originally developed to study the role of excitotoxicity following traumatic neuronal injury approximately three decades ago [24]. It was subsequently adopted to study other facets of the neuro-inflammatory response post-traumatic injury including astrogliosis [52,53] and axonal regeneration [54]. Whilst our findings demonstrate that the neuro-inflammatory response to mechanical injury was effectively captured with the use of this in vitro model, we acknowledge that extrapolation to TBI in the clinical setting must be cautiously considered. Indeed, TBI is heterogeneous both in terms of the mechanical forces involved and anatomical regions affected. Nonetheless, given that the protracted neuro-inflammatory response constituting “secondary” injury is thought to result in longer term morbidity, our findings that glycyrrhizin enhances neurogenesis post ICM-exposure is encouraging. Following injury, we demonstrated upregulation of HMGB1 gene expression and extracellular release, neuronal loss, microglial activation, cytoplasmic translocation of HMGB1 in neurons and microglia at earlier time points, and subsequent upregulation of mRNAs of other pro-inflammatory cytokines. These findings may be explained by the following sequence of events: (i) passive HMGB1 release from Tuj1^+^ neuronal death following mechanical injury; (ii) stimulation of microglia to adopt a pro-inflammatory phenotype with subsequent upregulation of HMGB1 gene expression and active secretion; and (iii) establishment of a pro-inflammatory HMGB1 positive feedback cycle resulting in up-regulation of other cytokines including TNF-α and NOS-2. Several in vitro studies indicate passive release of HMGB1 by dying cells following subarachnoid haemorrhage, ischaemic stroke, and other non-neurological pathologies [55,56,57,58]. Given the significant loss of Tuj1^+^ cells post-injury, our results suggest that dying neurons are a likely source of early HMGB1 release, but further studies examining co-localisation of cell death markers with Tuj1^+^HMGB1^+^ cells will be required to confirm this finding. At 24 h post-injury, both microglia and neurons demonstrated significant increase in the proportion of cytoplasmic HMGB1. This indicates that neurons may be responsible for an initial surge in HMGB1, followed by synergistic involvement of microglia and neurons at 24 h post-injury. This is consistent with current evidence demonstrating HMGB1 mediated microglial activation via the HMGB1/TLR4/NFkB axis in several disease contexts, including Parkinson’s disease, stroke, and epilepsy [59,60,61].

Our results demonstrate that ICM generated from mixed glial cultures did not reduce total cell count significantly but had a significantly negative impact on neurogenesis and astrogliosis. The fact that HMGB1 inhibition reversed the effects of ICM on neurogenesis indicates an anti-neurogenic effect of HMGB1 at the concentration levels reached post-injury. It is unclear whether this is due to increased NSPC death, reduced NSPC proliferation, or reduced NSPC differentiation. Further studies using immunolabelling to detect actively proliferating cells and dying cells will be required to elucidate this. Also, in vivo studies are required to examine whether HMGB1 inhibition results in longer term survival of mature newborn neurons post-injury, building on existing evidence that these neurons do not survive despite enhanced cortical neurogenesis post-TBI [13]. Previous studies have demonstrated involvement of HMGB1 with astrocytes following injury, and the development of cerebral oedema post-TBI has been attributed to this [16]. Our results demonstrate a negative impact of ICM on astrogliosis, which was unchanged by the addition of glycyrrhizin. We collected our ICM at 24 h post scratch injury, and it is possible that collection at different time points may have a different effect on astrocytes. Alternatively, given evidence that HMGB1 indirectly stimulates upregulation of aquaporin (AQP4) expression in astrocytes [62] resulting in pathologically increased permeability of the blood–brain–barrier, it may be possible that HMGB1 acts primarily on mature astrocytes following injury rather than enhancement of astrogliosis per se.

Despite increasing interest in promoting endogenous neurogenesis to enhance repair post-TBI, the effects of HMGB1 on NSPC post-injury have not been previously examined. One recent study evaluated the effect of HMGB1 on NSPCs using murine neurosphere cultures in the absence of injury [63]. Interestingly, HMGB1 exerted a positive effect on NSPC migration via RAGE at a concentration of 1 ng/mL. Following injury, however, a neurotoxic effect of HMGB1 is apparent. One study examined the effect of ICM collected from N-methyl-D-aspartate (NMDA)-treated primary cortical cultures on fresh primary cortical cultures as a model of post-ischaemic brain injury [64]. ICM resulted in significant neuronal apoptosis, an effect that was reversed by HMGB1 antagonism and shown to occur via HMGB1/RAGE-p38 MAPK/ERK signaling pathway. Therefore, we chose to examine the expression of RAGE in NSPC cultures as a target candidate for antagonism. We selected glycyrrhizin as the optimal antagonist for the following reasons: (i) increasing evidence implicates prevention of RAGE binding as its primary mode of HMGB1 antagonism [17]; (ii) it displays a relatively safe side effect profile, demonstrating its potential use in vivo; and (iii) studies have demonstrated beneficial effects following systemic administration in animal in vivo models of TBI [17,65]. However, the effects of glycyrrhizin may extend beyond RAGE antagonism, given evidence that it binds directly to HMGB1 at multiple binding sites (F37, A16, V19, R23 and K42) and prevents cytoplasmic translocation [66]. Whilst HMGB1 release post-injury is associated with pro-inflammatory consequences, an equal appreciation of potentially beneficial effects is essential. Indeed, before its recent recognition as a pro-inflammatory cytokine-like protein, HMGB1 was originally described as “amphoterin”, a protein responsible for enhancing neurite outgrowth [67,68,69]. The potential for negating beneficial effects of HMGB1 is illustrated by mixed results from some studies demonstrating delayed motor recovery and lack of neuroprotection with HMGB1 antagonism post-injury [70,71]. Therefore, the following must be clarified in the therapeutic approach: (i) concentration range at which HMGB1 is anti-neurogenic/ neurotoxic; and (ii) synergistic effects with pro- and anti-inflammatory cytokines post-injury.

## 5. Conclusions

For the first time, we have evaluated the effects of HMGB1 on cortical neurogenesis following mechanical injury. We demonstrate that: (i) canonical pathways of HMGB1 are activated following rodent needle cortical injury in vivo; (ii) needle scratch injury enhances microglial activation and neuronal loss in vitro; and (iii) HMGB1 exerts a detrimental but pharmacologically reversible effect on neurogenesis following exposure of NSPC cultures to injury condition media. Further in vivo studies are required to elucidate the potentially therapeutic effects of blocking the HMGB1-RAGE axis in promoting functional recovery post-TBI.

## Figures and Tables

**Table 1:** HMGB1 signaling pathway is upregulated at 2 h post injury. List of the differentially expressed genes in the HMGB1 canonical pathway. (Overlap *p* < 0.01, Z score 2.53, N: 4–5 per condition).

Symbol	Entrez Gene Name	Expr Log Ratio	Expr False Discovery Rate (q-Value)	Expression
CCL2	C-C motif chemokine ligand 2	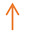 2.991	0.000265	Up
CLCF1	cardiotrophin like cytokine factor 1	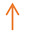 1.574	0.0353	
IL1R1	interleukin 1 receptor type 1	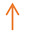 1.154	0.0275	Down
JUN	Jun proto-oncogene, AP-1 transcription factor subunit	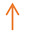 0.814	0.00194	Up
MAPK12	mitogen-activated protein kinase 12	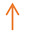 1.224	0.00468	Up
NGFR	nerve growth factor receptor	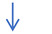 −2.276	0.0138	Up
PIK3C2G	phosphatidylinositol-4-phosphate 3-kinase catalytic subunit type 2 gamma	−2.42	0.0202	Up
RAC2	Rac family small GTPase 2	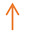 1.652	3.11E-05	
RAC3	Rac family small GTPase 3	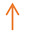 0.486	0.0391	
RALB	RAS like proto-oncogene B	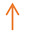 0.332	0.0212	Up
RASD2	RASD family member 2	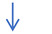 −1.83	1.85E-05	
RHOC	ras homolog family member C	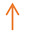 0.591	0.0137	Up
RHOH	ras homolog family member H	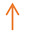 0.872	0.0282	Up
RRAS	RAS related	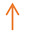 0.587	0.00878	Up
SERPINE1	serpin family E member 1	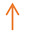 1.876	0.0198	
TGFB1	transforming growth factor beta 1	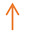 0.685	0.0194	Up
TNFRSF11B	TNF receptor superfamily member 11b	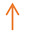 0.873	0.0488	Up
TNFRSF1A	TNF receptor superfamily member 1A	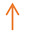 0.965	0.00707	Up
